# Characteristics and nutritional value of silkworm (*Bombyx mori*) pupae-fortified chicken bread spread

**DOI:** 10.1038/s41598-022-05462-x

**Published:** 2022-01-27

**Authors:** Supatra Karnjanapratum, Pensiri Kaewthong, Sylvia Indriani, Kantiya Petsong, Sirima Takeungwongtrakul

**Affiliations:** 1 School of Food Industry, King Mongkut’s Institute of Technology Ladkrabang, Bangkok, 10520 Thailand; 2 School of Management, Walailak University, Thasala, Nakhon Si Thammarat 80161 Thailand; 3 Food Technology and Innovation Research Centre of Excellence, Department of Agro-Industry, School of Agricultural Technology, Walailak University, Thasala, Nakhon Si Thammarat 80161 Thailand; 4 Department of Food Technology, Faculty of Technology, Khon Kaen University, Khon Kaen, 40002 Thailand; 5 Department of Agricultural Education, School of Industrial Education and Technology, King Mongkut’s Institute of Technology Ladkrabang, Bangkok, 10520 Thailand

**Keywords:** Proteins, Biophysical chemistry, Lipids, Natural products

## Abstract

This study aimed to apply silkworm pupae (SP) to food product development. The characteristics and sensory acceptance of chicken bread spread fortified with SP at different levels (0%; SP0, 25%; SP25, 50%; SP50, and 75%; SP75) were evaluated. The fat content of the bread spread was significantly increased, whereas the protein content was decreased with increasing levels of SP (*p* ≤ 0.05). The increased level of SP resulted in the final products being dark in color, as indicated by the significant decrease in L* and the significant increase in a* and b* (*p* ≤ 0.05). SP50 was accepted by the consumer. Thereafter, the characteristics and sensory acceptance of SP50 with different levels of coconut oil (CO) (100%; SP50-100, 70%; SP50-70, 40%; SP50-40, and 10%; SP50-10 of CO content in the control sample) were studied. The firmness and stickiness increased, whereas TEF decreased with decreasing CO levels, which was related to the decreased spreadability of SP50. SP50-40 obtained satisfactory sensory properties by the consumer. The energy value for SP50-40 was within the normal range for bread spread products. Therefore, SP could be a source of fat and protein for the production of an alternative food product to increase the added value of edible insects.

## Introduction

Edible insects are an alternative food source for consumers because they are a good source of proteins, fat, vitamins, minerals, and energy. Apart from nutrition, raising insects requires less land and water and has less impact on the environment and economy compared to livestock production. Many insect species are consumed, including crickets, grasshoppers, navel, and silkworm pupae^1^. Silkworm (*Bombyx mori*) pupae are a popular edible insect consumed in many areas, especially in Asia, including in Thailand, China, Indonesia, Vietnam, and Korea^2,3^. Silkworm pupae are by-products of the silk industry and can be a good source of protein and fat^4,5^. Rumpold and Schluter^1^ found that silkworm pupae (48.7% dry basis) had lower protein content than cricket (61.2% dry basis) and grasshoppers (62.5% dry basis), whereas silkworm pupae had a high fat content (30.1% dry basis). High levels of essential amino acids have been observed in silkworm pupae proteins, such as phenylalanine, methionine, and valine^5^. Liu et al.^6^ and Kotake-Nara et al.^7^ found that silkworm pupae oil is rich in α-linolenic acid (ALA), an essential fatty acid for the human diet. Furthermore, silkworm pupae contain an α-glucosidase inhibitor, 1-deoxynojirimycin (DNJ), which might reduce postprandial hyperglycemia and the absorption of carbohydrates^5^. Therefore, silkworm pupae are valuable sources of food products.

Spreadable products are normally spread onto foods such as bread and crackers to improve their flavor or texture. Several spreadable products are sold commercially, including cheese spread, mayonnaise, jam, jelly, peanut butter spread, liver pâté, and chicken meat spread^8–10^. Peanut butter spread^8^, liver pâtés^9^, and chicken meat spread^10^ are emulsion products that mainly consist of protein and fat.

The chicken meat spread^10^ was very interested in fortification with silkworm pupae based on the character of the product (emulsion products mainly consist of protein and fat) and the ingredient that might mask the unique flavor of silkworm pupae such as onion, ginger, and garlic. The chicken breast meat was healthy meat due to its high protein content (22.7%) and low fat content (1.3%)^11^ while silkworm pupae had high fat content (30.1% dry basis)^1^. However, silkworm pupae oil consisted of an essential fatty acid for the human diet as mentioned before^6,7^. Furthermore, silkworm pupae were also considered as new available sources of high-quality protein which consisted of the essential amino acid required for human health. The protein from silkworm pupae showed efficiently worked in antiapoptotic activity, hepatoprotective, wound dressings, anticancer agent, regulation of blood glucose and lipids, antigenotoxicity, etc.^12^.

The spreadability and emulsion stability properties of spreadable products are important and affect consumer acceptance. Oils and fats are the main components that affect the spreadability of spreadable products^13^. Many kinds of oils and fats are used in spreadable products, such as butter, margarine, and other fats of vegetable, animal, or marine origin^14^. Coconut oil is a potential ingredient for the development of food products because of its various health benefits and high stability. Coconut oil has a high content of medium-chain fatty acids (MCFAs), such as lauric acid (major fatty acid), caprylic acid, and capric acid^15^. MCFAs can decrease the risk of atherosclerosis and heart disease^16^. Therefore, the development of chicken bread spread fortified with silkworm pupae and coconut oil could be an alternative product for consumers.

The aim of this study was to develop silkworm pupae-fortified chicken bread. The suitability of silkworm pupae and coconut oil levels for producing silkworm pupae-fortified chicken bread spread were evaluated. Silkworm pupae-fortified chicken bread spread was evaluated in terms of chemical composition, physical characteristics, sensory properties, and nutritional value.

## Materials and methodology

### Materials

Frozen silkworm (*Bombyx mori*) pupae (SP) were obtained from a supermarket in Bangkok, Thailand. The silkworms were thawed at 4 ± 2 °C until the core temperature of the samples reached 0–4℃ for use. Fresh chicken breast, coconut oil, tapioca flour, vinegar, salt, pepper powder, and condiments were procured from a local market in Ladkrabang, Bangkok, Thailand.

### Experimental design

**Figure 1:**
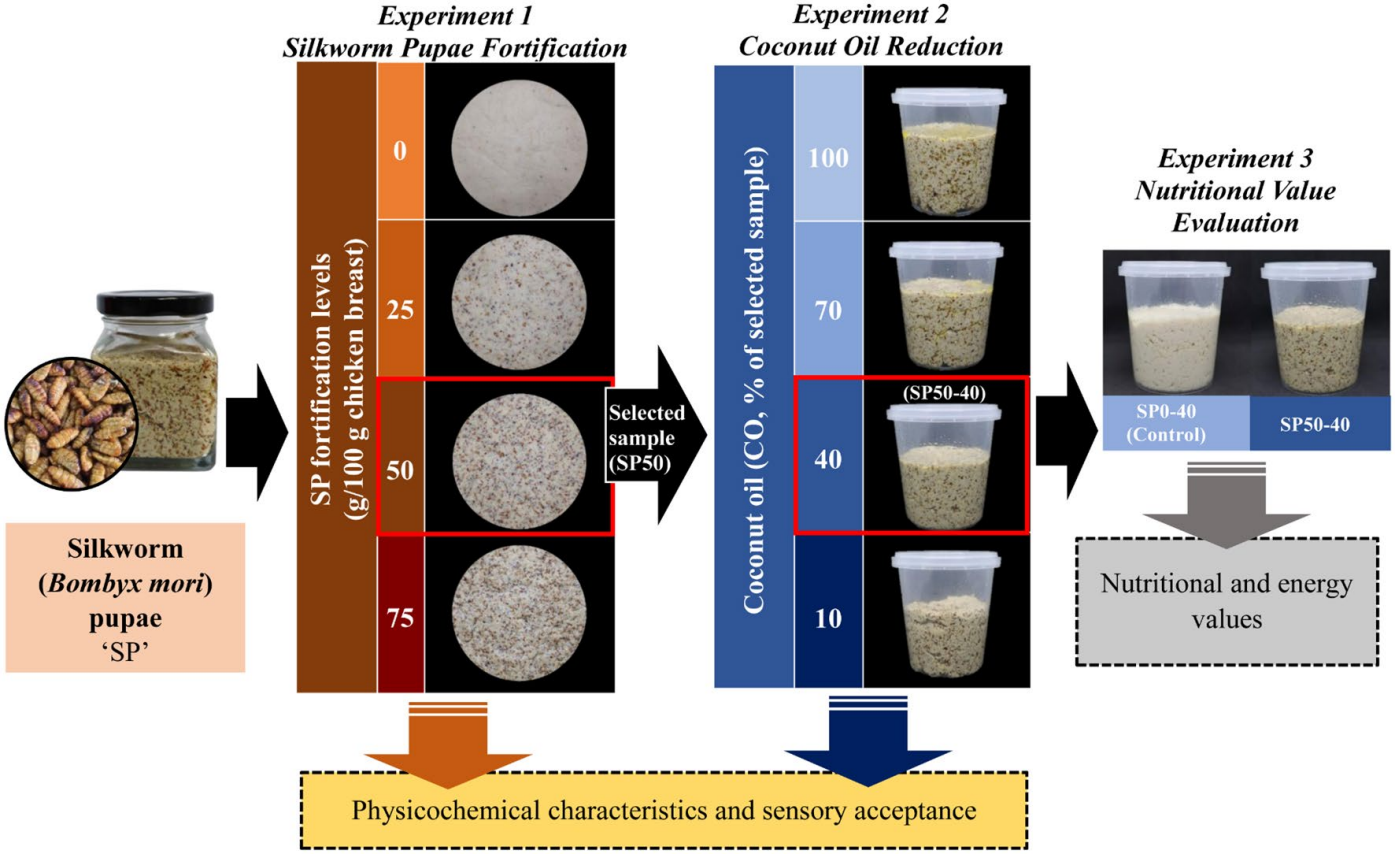
Scheme of experimental design for development of silkworm (*Bombyx mori*) pupae-fortified chicken bread spread on characteristics and nutritional value.

Experiment 1: Development of silkworm pupae-fortified chicken bread spread (Fig. 1).

The ingredients used to prepare SP-fortified chicken bread are presented in Table 1. SP were cooked by blanching in boiling water (98–99 °C) for 5 min and were then drained for 2 min. Thereafter, the cooked SP were blended using a blender (Mara, MR-1268, Thailand) for 1 min. Fresh chicken breasts were blended and blanched using the same process described for the silkworm pupae. All ingredients were mixed and blended for 5 min, except for vinegar. The mixed sample was pasteurized by controlling the core temperature of the sample at 73 ± 2 °C and holding for 15 min. Vinegar was added after the pasteurization process. SP-fortified chicken bread spread was blended again in a sterile blender and packed in a sterile glass bottle. Then, the samples were kept at ambient temperature until the core temperature reached 23 ± 2 °C, and the chemical composition, pH, color value (L*, a*, b*), textural properties, emulsion stability, and sensory characteristics were analysed.

**Table 1:** Ingredients were used to prepare silkworm pupae-fortified chicken bread spread.

Ingredients	Amount (g)			
	SP0 (Control)	SP25	SP50	SP75
Chicken breast	250.0	62.5	125.0	187.5
Silkworm pupae	–	187.5	125.0	62.5
Coconut oil	52.5	52.5	52.5	52.5
Condiments*	13.0	13.0	13.0	13.0
Tapioca flour	18.0	18.0	18.0	18.0
Vinegar	9.0	9.0	9.0	9.0
Salt	2.0	2.0	2.0	2.0
Sugar	1.0	1.0	1.0	1.0
Pepper powder	1.0	1.0	1.0	1.0
Water	13.0	13.0	13.0	13.0

Source: Modified from Arya et al.^10^.

*Onion:ginger:garlic (3:2:1).

SP0 (Control): Chicken bread spread without silkworm pupae fortification.

SP25, SP50, and SP75: Silkworm pupae-fortified chicken bread spread with the replacement of chicken meat at 25, 50, and 75%, respectively.

Experiment 2: Effect of coconut oil reduction on the characteristics and chemical composition of silkworm pupae-fortified chicken bread spread (Fig. 1).

Chicken bread spread fortified with 50% SP (the optimum SP level obtained from experiment 1) was prepared using the same ingredients and process as described for experiment 1, except for the level of coconut oil (CO). The samples were prepared using 70% (SP50-70), 40% (SP50-40), and 10% (SP50-10) CO content in the control sample (100% CO; SP50-100). The chemical composition, pH, color value (L*, a*, b*), textural properties, emulsion stability, and sensory characteristics of the samples with different CO levels were evaluated.

Experiment 3: Nutritional value of silkworm pupae-fortified chicken bread spread (Fig. 1).

The nutritional and energy values of chicken bread spread fortified with 50% of SP and decreased CO content from the recipe at 40% (the optimum CO level obtained from experiment 2; SP50-40) were determined and compared with that of without the fortification of silkworm pupae.

### Analyzes

#### Chemical compositions

The chemical composition of SP and SP-fortified chicken bread spread was determined using an oven method for moisture content; the Kjeldahl method for protein content; the Soxhlet solvent extraction for fat content; and a furnace for ash content following AOAC methods^17^. Thereafter, the carbohydrate content was calculated by the difference between 100 and the sum of moisture, protein, fat, and ash content^4^.

#### Water activity (a_w_), pH, and color

The *a*_*w*_ was measured using a water activity meter (Aqua Lab 4TE, Decagon Devices, USA). The pH of minced SP, minced chicken breast meat and SP-fortified chicken bread spread was measured by diluting with distilled water (1:5, w/v), followed by homogenization, then measured using a pH meter (FEP20-FiveEasy Plus, Mettler Toledo, Switzerland)^18^. The pH of CO was determined using litmus paper.

Color of samples was measured using a HunterLab colorimeter (HunterLab, ColorQuest XE, USA) with a 1-inch port size, 10° observers, and illuminant D65. Briefly, samples were placed in a cuvette and recorded using the CIE color system, as L * (lightness), a* (redness), and b ∗ (yellowness). The total difference in color (ΔE ∗) of sample was calculated by comparing with the control of each experiment, as described by Karnjanapratum and Benjakul^19^.

#### Textural properties

Textural properties of sample were measured following the method tailored by Rezler et al.^20^ with slight modification. Briefly, the sample (60 g) was placed in a 100 mL beaker, and the sample was compressed with a spoon until all samples were at the same height. Firmness (N), spreadability (N·s), and stickiness (N) of the sample were analyzed using a texture analyzer (TA.XT.plus®, Texture Technologist Corp., USA) equipped with a cone probe (60° conical probe perspex; P/60C) and a 50 kg load cell. The pre-test speed, test speed, and post-test speed of the measurement were set at 1, 2, and 10 mm/s, respectively. The probe penetrated the test sample at 20 mm. The maximum force of the first curve, the area under the first curve, and the maximum force of the second curve on the opposite side of the first curve refer to the firmness (N), spreadability (N·s), and stickiness (N), respectively.

#### Emulsion stability

The emulsion stability of the samples was evaluated following the method described by Martin et al.^21^. Samples (5 g) were placed in a tube, centrifuged (4,000 rpm, 20 min, 25 °C, Eppendorf 5804R, Germany), and the supernatant was removed. The remaining oil spread over the walls of the tube was removed by rinsing with 2 mL of hexane. The pellet was weighed. The percentage of total expressible fluid (TEF) was calculated using the following equation:

$${\text{\% TEP = [(Weight}}\;{\text{of}}\;{\text{tube}}\;{\text{and}}\;{\text{sample}}{ - }{\text{Weight}}\;{\text{of}}\;{\text{tube}}\;{\text{and}}\;{\text{pellet)/Weight}}\;{\text{ of}}\;{\text{ sample] }}\times 100\%$$

### Sensory evaluation

The sensory attributes of the sample were evaluated by 30 untrained panellists. The liking score of the sample was evaluated using a 9-point hedonic scale (1 = dislike extremely, and 9 = like extremely) based on the appearance, color, texture, taste, after taste, and overall acceptance.

### Nutrition and energy values

Moisture, fat, protein, and ash content, were analyzed using AOAC methods^17^. The total carbohydrate content was calculated by the difference between 100 and the sum of moisture, protein, fat, and ash^4^. The total fiber, total sugar, and cholesterol were determined following the AOAC method using method numbers 985.29, 925.35, and 976.26, respectively^17^. AOAC method number 984.27^17^ was used to analyze the sodium and calcium contents of the samples. The energy value of the sample was evaluated following Sullivan and Carpenter^22^.

### Statistical analysis

Experiments were run in triplicate. The physicochemical characteristics of the sample were analyzed using a completely randomized design (CRD). A randomized complete block design (RCBD) was used for sensory evaluation. A pairwise T-test was applied to compare the nutritional value between two samples using the SPSS program (IBM SPSS Statistics, IBM, New York, USA). Furthermore, the pH of SP and chicken breast was also analyzed using a pairwise T-test. Significant differences between the means were calculated using Duncan’s multiple range test at a 95% confidence level.

## Result and discussion

### Development of silkworm pupae-fortified chicken bread spread

The chemical compositions of SP-fortified chicken bread spread at 0% (Control; SP0), 25% (SP25), 50% (SP50), and 75% (SP75) are shown in Table 2. The replacement of chicken breast meat with SP affected the chemical composition of chicken bread spread, especially the protein and fat content. The protein and moisture contents of the samples were significantly decreased (*p* ≤ 0.05); in contrast, fat and carbohydrate contents were significantly increased (*p* ≤ 0.05) with increasing levels of SP. The ash content of the sample increased slightly with increasing levels of SP. The difference in the chemical composition of SP and chicken meat influenced the change in chemical composition of the SP-fortified chicken bread spread. The protein, fat, moisture, ash, and carbohydrate contents of SP were 15.79 ± 0.01, 9.69 ± 0.02, 72.33 ± 0.92, 1.26 ± 0.00, and 1.59 ± 0.47%, respectively. The chicken breast meat had a higher protein content (22.7%) and a lower fat content (1.3%)^11^ compared to SP. The results of the current study were consistent with those of Park et al.^3^, who found that the protein content of meat batter decreased, whereas fat content increased with increasing levels of SP powder. The use of SP for preparing chicken breast spread also affected the carbohydrate content of the final products, and Mishra et al.^4^ reported that SP had a carbohydrate content of 1.2–1.8% depending on the silkworm species. The major carbohydrates in insects are chitin and glycogen^23^. Chitin, an insoluble carbohydrate, is found in SP exoskeletons^12,24^. The chemical composition of SP-fortified chicken bread spread was considered as an emulsion product consisting mainly of protein and fat.

**Table 2:** Physicochemical characteristics of silkworm pupae-fortified chicken bread spread.

Characteristics	SP0 (control)	SP25	SP50	SP75
**Chemical composition**				
Protein*	15.63 ± 0.22^a^	14.56 ± 0.24^b^	13.64 ± 0.32^c^	12.12 ± 0.07^d^
Fat	12.41 ± 0.08^d^	15.25 ± 0.16^c^	16.06 ± 0.21^b^	16.49 ± 0.18^a^
Moisture	64.81 ± 1.43^a^	61.71 ± 0.94^b^	59.99 ± 0.66^c^	58.07 ± 0.37^d^
Ash	1.51 ± 0.04^b^	1.52 ± 0.03^b^	1.59 ± 0.06^b^	2.18 ± 0.08^a^
Carbohydrate	5.20 ± 0.14^c^	7.55 ± 0.69^b^	8.71 ± 0.67^b^	11.14 ± 0.61^a^
Water activity (*a*_*w*_)	0.985 ± 0.004^b^	0.989 ± 0.003^ab^	0.991 ± 0.002^a^	0.992 ± 0.001^a^
pH	5.38 ± 0.12^a^	5.59 ± 0.13^a^	5.55 ± 0.13^a^	5.44 ± 0.01^a^
**Color**				
L*	72.58 ± 0.10^a^	63.06 ± 0.07^b^	58.21 ± 0.03^c^	53.53 ± 0.34^d^
a*	0.36 ± 0.03^d^	1.06 ± 0.06^c^	1.75 ± 0.02^b^	2.17 ± 0.23^a^
b*	13.64 ± 0.13^d^	16.12 ± 0.07^c^	16.35 ± 0.25^b^	16.60 ± 0.21^a^
∆E*	–	48.68 ± 0.18^c^	107.97 ± 4.77^b^	187.54 ± 4.71^a^
**Textural properties**				
Firmness (N)	2.45 ± 0.29^a^	2.16 ± 0.10^b^	1.67 ± 0.10^c^	1.67 ± 0.10^c^
Stickiness (N)	0.45 ± 0.05^a^	0.42 ± 0.01^b^	0.34 ± 0.02^c^	0.34 ± 0.01^c^
Spreadability (N·s)	15.20 ± 1.77^a^	13.73 ± 0.39^b^	10.79 ± 0.29^c^	10.79 ± 0.39^c^

Data are expressed as mean ± standard deviation (n = 3; n = 6 for color and textural properties).

*The conversion factor used is 6.25.

SP0 (Control): Chicken bread spread without silkworm pupae fortification.

SP25, SP50, and SP75: Silkworm pupae-fortified chicken bread spread with the replacement of chicken meat at 25, 50, and 75%, respectively.

Different lowercase superscripts within the same row indicate significant differences (*p* ≤ 0.05).

The characteristics of SP-fortified chicken bread at different levels are presented in Table 2. The results showed that the *a*_*w*_ of chicken bread spread fortified with SP was slightly higher than that of the control sample. SP levels had no significant effect on the pH of the final product because there were no significant differences in pH between chicken breast (5.68 ± 0.11) and SP (6.21 ± 0.05). The *a*_*w*_ and pH of the SP-fortified chicken bread spread were within the ranges 0.985–0.992 and 5.38–5.59, respectively. Most foods with *a*_*w*_ higher than 0.95 are suitable for the growth of bacteria, yeast, and mold^25^. Therefore, the proper storage of SP-fortified chicken bread spread should be considered.

**Figure 2:**
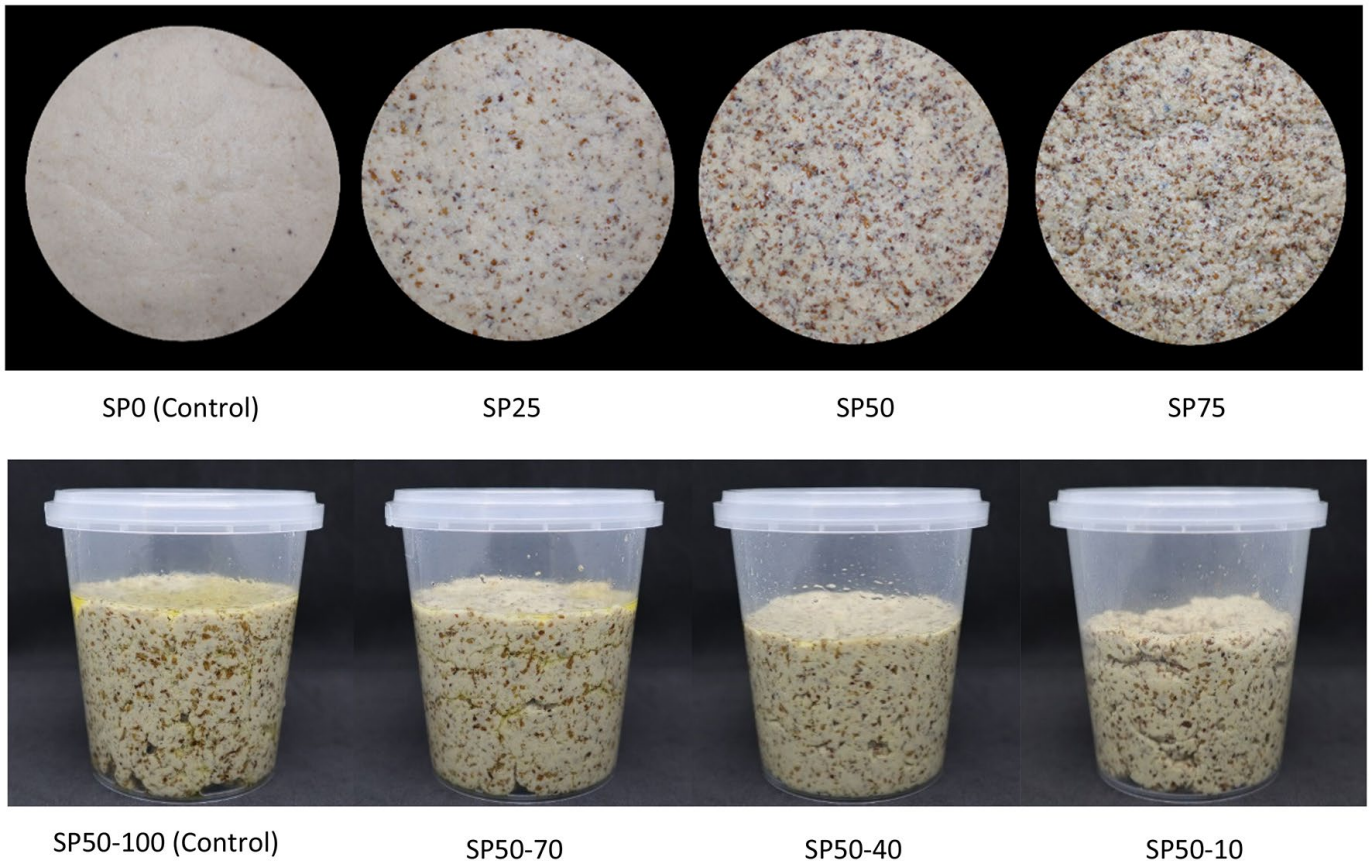
The characteristics of chicken bread spread-fortified with silkworm pupae. SP0, SP25, SP50, and SP75: Silkworm pupae-fortified chicken bread spread with the replacement of chicken meat at 0% (Control for experiment 1; without fortification), 25, 50, and 75%, respectively. SP50-100, SP50-70, SP50-40, and SP50-10: Chicken bread spread with 50% replacement of chicken meat by silkworm pupae using 100% (Control for experiment 2), 70, 40, and 10% of coconut oil in control sample of experiment 1, respectively.

The color value of the bread spread significantly changed with increasing levels of SP. Increasing levels of SP resulted in the final products presenting a dark color, as indicated by a significant increase in a* and b* and a significant decrease in L* (*p* ≤ 0.05). SP75 had the lowest L* (*p* ≤ 0.05). The dark color of SP-fortified chicken bread spread increased the total difference in color (ΔE ∗) compared to the control. The appearance and color of SP-fortified chicken bread spread at different levels are presented in Fig. 2. The change in color of the bread spread was due to the dark color of the SP exoskeleton (L* = 38.71 ± 0.03; a* = 6.68 ± 0.19; b* = 8.12 ± 0.12) compared to that of the chicken breast (L* = 67.24 ± 0.82; a* = 3.34 ± 0.07; b* = 17.59 ± 0.48). The results of the current study were similar to those of a previous study on SP powder-fortified emulsion meat products^3,24^. Those authors reported that the lightness (L*) of emulsion meat products decreased with increasing levels of SP powder.

Regarding texture evaluation of bread spread, firmness and stickiness decreased with increasing levels of SP (*p* ≤ 0.05) (Table 2). SP75 presented the lowest firmness and stickiness, significantly (*p* ≤ 0.05). Spreadability reflects the shear resistance during compression of the bread spread. The amount of work compressing decreased with increasing levels of SP, leading to increased spreadability of SP-fortified chicken bread spread. The reduced firmness and stickiness of bread spread are related to an increase in spreadability. The spreadability of bread ranged from 15.20 N·s at 0% SP to 10.79 N·s at 75% SP. Pearson and Gillett^26^ reported that fat and oil provide lubrication properties in emulsion products. Thus, the higher fat content of SP (9.69 ± 0.02%) compared to chicken breast meat (1.3%)^11^ might account for the decreased firmness and stickiness and increased spreadability of bread spread. Wagener and Kerr^27^ also found that firmness increased with increasing oil content, leading to reduced spreadability of nut butter. The spreadability was 0.51 and 47.86 N for nut butter containing 70 and 50% oil, respectively.

**Figure 3:**
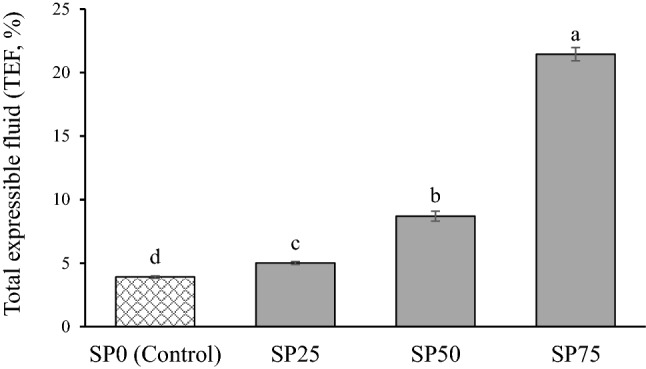
Total expressible fluid (TEF, %) of silkworm pupae-fortified chicken bread spread at different levels. SP0 (Control): Chicken bread spread without fortification with silkworm pupae. SP25, SP50, and SP75: Silkworm pupae-fortified chicken bread spread with the replacement of chicken meat at 25, 50, and 75%, respectively. Different letters on the bars indicate significant differences (*p* ≤ 0.05).

The impact of different levels of SP on the total expressible fluid (%TEF) of chicken bread spread is presented in Fig. 3. The percentage of TEF in the sample significantly increased with increasing levels of SP (*p* ≤ 0.05). The results indicated that the emulsion stability of bread spread was reduced by the increased level of SP, which was related to reduced firmness and stickiness (Table 2). The addition of SP led to an increase in fat content, whereas protein content, which worked as an emulsifier, was reduced in the final product. These results were consistent with those of Choi et al.^28^, who found that the replacement of lean pork meat with yellow mealworm increased the total expressible fluid separation and fat separation, leading to a reduction in the emulsion stability of frankfurters. Furthermore, Youssef and Barbut^29^ reported that the preparation of meat batter with a low level of protein caused low emulsion stability of the final product, as indicated by the high fluid loss during cooking. SP-fortified chicken bread spread is an oil-in-water emulsion product. As the matrix of emulsion meat, the denaturation and gelation of myofibrillar protein from chicken meat is the major component of the continuous phase, whereas oil droplets are a dispersed phase^30^. Normally, proteins in the emulsion system function as emulsifiers and prevent the coalescence of oil droplets^31^. The decrease in protein level and increase in fat level in the emulsion system caused the coalescence of oil droplets, leading to a reduction in emulsion stability^32^. Moreover, the SP included a hard exoskeleton with a mass exceeding 1%. A major component of the SP exoskeleton is chitosan, which is water-insoluble^12,24^ and may interrupt the emulsion system of the bread spread. Therefore, SP fortification resulted in the low emulsion stability of chicken breast spread, which reduced denseness and resistance during spreading, leading to an increase in the spread of bread spread product.

**Figure 4:**
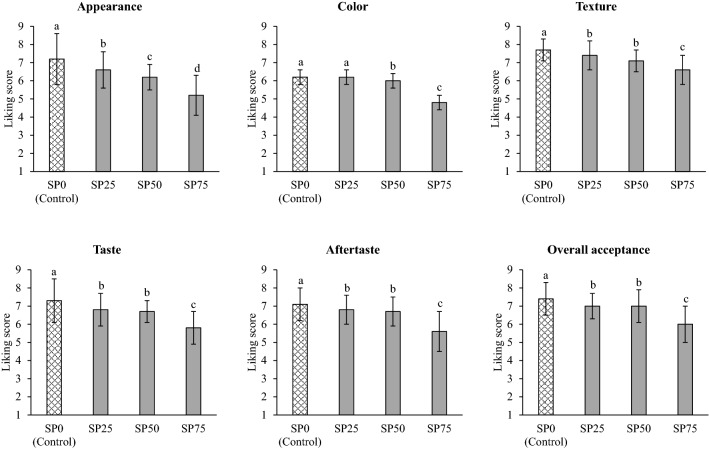
Sensory characteristics (liking score) of silkworm pupae-fortified chicken bread spread at different levels. SP0 (Control): Chicken bread spread without fortification with silkworm pupae. SP25, SP50, and SP75: Silkworm pupae-fortified chicken bread spread with the replacement of chicken meat at 25, 50, and 75%, respectively. Different letters on the bars indicate significant differences (*p* ≤ 0.05).

The sensory characteristics of SP-fortified chicken bread at different levels are presented in Fig. 4. The results showed that the increased level of SP fortification influenced the reduction of liking score for all the sensory attributes, especially at the highest fortification level (75%; *p* ≤ 0.05). The sensory characteristics of bread spread were related to changes in color and textural properties, as previously explained (Table 2). The lowest liking score for taste and aftertaste attributes was found for the bread spread with the highest level of SP fortification (SP75), probably due to the off-flavor of SP. Mishyna et al.^33^ reported that variations in the intensities of volatile profiles were found in SP, including alcohols, alkanes, aldehydes, esters, and ketones. Dimethyl disulfide, 2-methylbutanal, 3-methylbutanal, hexanal, and acetic acid were the common volatiles detected in raw SP. Two notable volatiles in the raw SP were 2-methylbutanal and 3-methylbutanal. Regarding the sensory evaluation, the common odor of raw SP has previously been described as ‘herbal’, ‘bean’, ‘fruity’, and ‘nutty’^33^. The off-flavor may also be affected by the high fat content of SP, because the volatile compounds responsible for the off-odors might comprise the fat part of pupae. Delicato et al.^34^ reported that insect fat resulted in baked products presenting a bad and enduring off-flavor, aftertaste, and rancid aroma. Defatting could reduce insect off-odors by eliminating undesirable volatile compounds and enhancing good odors^35^. Therefore, the unique flavor of SP caused a decrease in the liking score of the final food product. The results of the present study were consistent with those of Hirunyophata et al.^36^, who found that an increased level of SP powder affected the reduction of liking score in the breakfast cereal product. Moreover, SP75 presented the lowest sensory score for overall acceptance (*p* ≤ 0.05), whereas the control sample presented the highest sensory score for overall acceptance. No significant differences in the sensory scores for texture, taste, aftertaste, and overall acceptance were observed between SP25 and SP50. SP50 presented a liking score higher than 6 for all sensory attributes. Particularly, the liking score of chicken bread spread fortified with SP at 50% was higher than 7 for texture and overall acceptance. A liking score of 6 (liking slightly) on a 9-point hedonic scale was applied to indicate the acceptance of a food product by the consumer^36,37^. The sensory characteristics indicated that the fortification of chicken bread spread with 50% SP was accepted by the consumer. However, some panellists suggested that although this product was very easy to spread on the bread, there was oil separation. Zayas^32^ stated that emulsion stability was not dependent only on the protein stabilizer, but was also affected by the type and concentration of oil/fat. The oil droplets (discontinuous phase) remained close in the emulsion system when the concentration of oil was increased. This phenomenon caused the coalescence of a single oil droplet with other droplets, leading to reduced emulsion stability. Thus, the effects of CO reduction on the characteristics and chemical compositions were evaluated to determine a suitable level of CO for preparing SP-fortified chicken bread spread.

### Effect of coconut oil reduction on the characteristics of silkworm pupae-fortified chicken bread spread

The chemical composition of chicken bread spread fortified with 50% SP (SP50) with different CO levels (100%; SP50-100, 70%; SP50-70, 40%; SP50-40, and 10%; SP50-10 of CO content in the control sample) are presented in Table 3. The fat content of SP50 was significantly decreased, whereas the moisture, protein, and ash contents were significantly increased with reduced levels of CO (*p* ≤ 0.05). In contrast, no significant differences in the carbohydrate content of SP50 were observed with different CO levels. The change in the fat/oil content affected the chemical composition of the final food product^38,39^. A previous study also found that the protein and moisture contents of sausages were significantly increased with reduced levels of fat. Reduced fat levels also affect the color, *a*_*w*_, weight loss, and sensory characteristics of sausages^38^.

**Table 3:** Physicochemical characteristics of chicken bread spread-fortified with 50% of silkworm pupae with different levels of coconut oil.

Characteristics	SP50-100 (Control)	SP50-70	SP50-40	SP50-10
**Chemical composition**				
Protein*	15.22 ± 0.66^b^	15.29 ± 0.11^b^	17.15 ± 0.62^a^	17.91 ± 0.66^a^
Fat	17.82 ± 0.14^a^	14.28 ± 0.12^b^	9.96 ± 0.10^c^	5.92 ± 0.06^d^
Moisture	54.50 ± 0.18^d^	57.44 ± 0.42^c^	59.90 ± 0.94^b^	62.36 ± 0.19^a^
Ash	1.36 ± 0.01^c^	1.36 ± 0.01^c^	1.51 ± 0.01^b^	1.61 ± 0.01^a^
Carbohydrate	11.10 ± 0.56^a^	11.63 ± 0.43^a^	11.49 ± 0.84^a^	12.18 ± 0.58^a^
Water activity (*a*_*w*_)	0.993 ± 0.003^ab^	0.994 ± 0.004^a^	0.989 ± 0.001^ab^	0.988 ± 0.003^b^
pH	5.64 ± 0.07^d^	5.95 ± 0.02^c^	6.04 ± 0.02^b^	6.18 ± 0.02^a^
**Color**				
L*	57.89 ± 0.52^d^	59.53 ± 0.24^c^	60.25 ± 0.03^b^	62.09 ± 0.33^a^
a*	1.82 ± 0.04^a^	1.44 ± 0.22^b^	1.48 ± 0.11^b^	1.01 ± 0.16^c^
b*	17.10 ± 0.12^a^	16.31 ± 0.42^b^	16.23 ± 0.09^b^	15.55 ± 0.28^c^
∆E*	–	1.83 ± 0.32^b^	3.34 ± 1.13^b^	10.71 ± 3.36^a^
**Textural properties**				
Firmness (N)	1.67 ± 0.10^c^	1.77 ± 0.20^c^	3.04 ± 0.29^b^	5.10 ± 0.49^a^
Stickiness (N)	0.32 ± 0.02^c^	0.34 ± 0.02^c^	0.59 ± 0.07^b^	0.87 ± 0.10^a^
Spreadability (N·s)	10.79 ± 0.69^c^	10.98 ± 0.98^c^	18.44 ± 0.98^b^	31.77 ± 2.16^a^

Data are expressed as mean ± standard deviation (n = 3; n = 6 for color and textural properties).

*The conversion factor used is 6.25.

SP50-100 (Control): Chicken bread spread with 50% replacement of chicken by silkworm pupae without fat reduction (100% coconut fat).

SP50-70, SP50-40, and SP50-10: Chicken bread spread with 50% replacement of chicken by silkworm pupae using 70, 40, and 10% coconut oil in the control sample, respectively.

Different lowercase superscripts within the same row indicate significant differences (*p* ≤ 0.05).

Table 3 presents the characteristics of chicken bread spread fortified with 50% SP at different CO levels. The *a*_*w*_ of SP50 decreased slightly with reduced CO levels; however, changes in the pH, L*, a*, b*, firmness, stickiness, and spreadability were observed. The *a*_*w*_ of SP50 ranged from 0.988 to 0.993. The pH of SP50 was significantly increased with reducing levels of CO (*p* ≤ 0.05) because the pH of CO (5.75) was lower than that of SP (6.41 ± 0.05) and was similar to chicken breast (5.62 ± 0.01).

The L* increased with decreasing CO levels, whereas a* and b* decreased (*p* ≤ 0.05). This result led to an increase in the total color difference (ΔE*) in SP50 with CO reduction compared to the control sample (without CO reduction). The highest total difference in color (ΔE*) compared to the control sample was observed with SP50-10. The change in color of SP50 with differing CO levels was probably due to differences in oil separation. There was less oil separation in SP50, with the highest reduction in CO level (SP50-10), as shown in Fig. 2.

The firmness (N) and stickiness (N) of SP50 increased significantly with decreasing levels of CO (*p* ≤ 0.05). Notably, SP50-10 presented the highest firmness and stickiness (*p* ≤ 0.05) and the lowest spreadability. This result is similar to that obtained by Hand et al.^40^, who observed a higher shear resistance in low-fat frankfurters than in high-fat frankfurters. Aydın and Özdemir^41^ stated that hydrogenated palm oil could be used to reduce firmness and improve the spreadability of carob-flour-based functional spread. Therefore, the firmness of carob-flour-based functional spread prepared with a low level of hydrogenated palm oil was higher than that prepared with a high level of hydrogenated palm oil. The results of the present study imply that reduced CO levels increased denseness and resistance during spreading. High spreadability was observed in the sample with a high CO level due to the fat and oil providing lubrication properties in emulsion products^26^. The friction coefficient of emulsion-filled gels gradually decreases with increasing oil concentration^42^. Therefore, the reduced oil level affected the characteristics of chicken bread spread fortified with SP, especially color and textural properties.

**Figure. 5:**
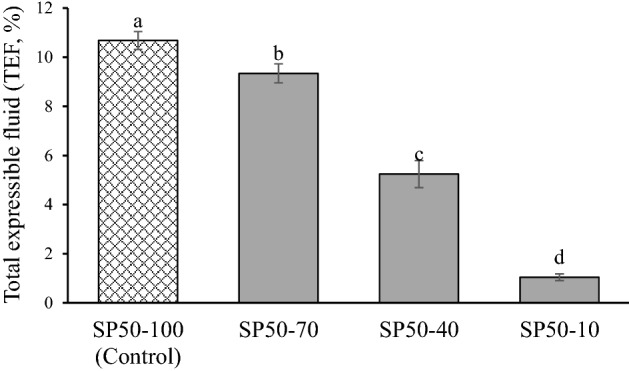
Total expressible fluid (TEF, %) of chicken bread spread-fortified with 50% of silkworm pupae with different levels of coconut oil. SP50-100 (Control): Chicken bread spread with 50% replacement of chicken by silkworm pupae without fat reduction (100% of coconut fat). SP50-70, SP50-40, and SP50-10: Chicken bread spread with 50% replacement of chicken meat by silkworm pupae using 70, 40, and 10% of coconut oil in control sample, respectively. Different letters on the bars indicate significant differences (*p* ≤ 0.05).

**Figure 6:**
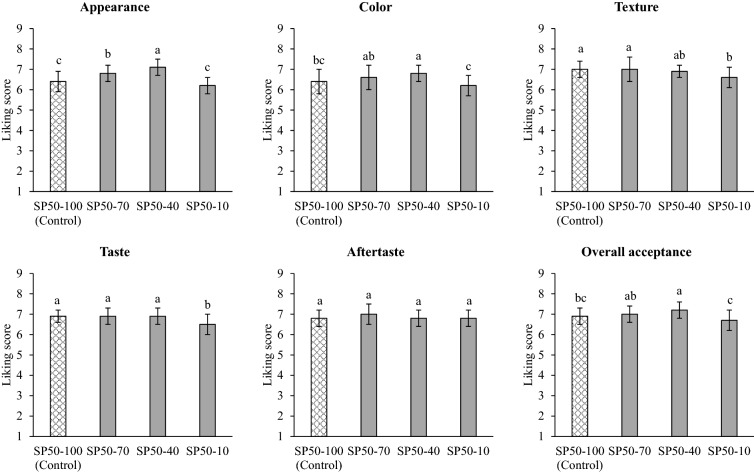
Sensory characteristics (liking score) of chicken bread spread-fortified with 50% of silkworm pupae with different levels of coconut oil. SP50-100 (Control): Chicken bread spread with 50% replacement of chicken by silkworm pupae without fat reduction (100% of coconut fat). SP50-70, SP50-40, and SP50-10: Chicken bread spread with 50% replacement of chicken meat by silkworm pupae using 70, 40, and 10% of coconut oil in control sample, respectively. Different letters on the bars indicate significant differences (*p* ≤ 0.05).

The total expressible fluid (%TEF) results for SP50 with different CO levels are shown in Fig. 5. The significant decrease in TEF in SP50 was affected by decreased CO levels (*p* ≤ 0.05). The characteristics of SP50 with different CO levels are shown in Fig. 2. There was no visible oil release when the CO levels were reduced until the sample contained 10% CO in the control sample (SP50-10). The reduction in TEF was related to the increased firmness and stickiness of bread spread, whereas spreadability was reduced (Table 3). The reduced CO levels also affected the sensory characteristics of SP50. The liking score for appearance, color, and overall acceptance tended to increase as the level of CO in the SP50 was reduced, except for the SP50-10 sample (Fig. 6). The reduction of CO to 10% in the control sample (SP50-10) was unacceptable, as indicated by the lowest liking score for all sensory attributes. Pearson and Gillett^26^ stated that fat in emulsion products provided a mouthfeel, which was described using terms such as creaminess, viscosity, body, lubricity, juiciness, smoothness, and texture. Therefore, the fat/oil content in emulsion meat products influences the acceptance score for the consumer. The SP50-40 sample presented the highest liking score for all attributes, and the score for appearance and overall acceptance was higher than 7. Thus, 40% CO content in the control sample was suitable for preparing SP50 with satisfactory sensory properties.

### Nutritional value of silkworm pupae-fortified chicken bread spread

Chicken bread spread fortified with SP (SP50-40) was prepared using chicken breast (125.0 g), SP (125.0 g), CO (21 g), condiments (13.0 g; onion:ginger:garlic at 3:2:1), tapioca flour (18.0 g), vinegar (9.0 g), salt (2.0 g), sugar (1.0 g), pepper powder (1.0 g), and water (13.0 g). Thereafter, the nutritional value of SP50-40 was evaluated and compared with that of chicken bread spread without fortification of SP using the optimum CO level obtained from experiment 2 (control). The nutritional values (chemical composition and energy value) of both samples are presented in Table 4. The SP50-40 sample contained a lower protein content (18.8 g/100 g) and a higher fat content (10.6 g/100 g) than the control sample (protein 20.6 g/100 g and fat 8.2 g/100 g), which was related to the chemical composition of SP (experiment 1). SP contained a higher fat content and lower protein content than chicken breast meat. Protein and fat are the main nutritional components in SP-fortified chicken bread spread. Tomotake et al.^5^ stated that SP (*Bombyx mori*) are alternative sources of high-quality proteins and lipids. SP oil consists of an essential fatty acid, α-linolenic acid (ALA)^6,7^. High levels of essential amino acids have been observed in SP proteins, including methionine, phenylalanine, and valine^5^. Furthermore, SP contains an α-glucosidase inhibitor, 1-deoxynojirimycin (DNJ), which may reduce postprandial hyperglycemia and the absorption of carbohydrates^5^. The fortification of chicken bread spread with SP also affected the carbohydrate and total fiber contents, which were higher in the SP50-40 sample than in the control sample. The major carbohydrates in insects are chitin and glycogen^23^. The SP exoskeleton consists of chitin, which is an insoluble carbohydrate^12,24^. Previous research reported that SP consisted of chitin approximately 3–4% in dry matter^43,44^. No significant differences were observed in the ash content of either sample. Sodium and calcium contents were higher in the control sample than in the SP50-40 sample. Conversely, the SP50-40 sample contained higher cholesterol (89 mg/100 g) and energy (203.6 kcal/100 g) values compared to the control sample (cholesterol 68.4 mg/100 g and energy value 180.9 kcal/100 g). Akande et al.^45^ reported that the energy value of SP powder is mainly obtained from a high fat composition. However, the SP50-40 sample contained lower cholesterol levels compared to the sheep liver pâté (115.1 mg/100 g) reported by Amaral et al.^46^. The energy value of SP50-40 was less than liver pâtés (371.7 kcal/100 g)^47^ and peanut butter spread (600.0–614.1 kcal/100 g)^48^. The energy value of sandwich spread (pork or beef) commonly consumed in the United States was reported to be 233 kcal/100 g^49^, which was higher than the energy value of chicken bread spread fortified with SP (SP50-40) in the present study.

**Table 4:** Nutrition value of chicken bread spread and chicken bread spread-fortified with silkworm pupae.

Parameters	SP0-40 (Control)	SP50-40
Protein (g/100 g)	20.55 ± 0.47^a^	18.83 ± 0.41^b^
Fat (g/100 g)	8.23 ± 0.01^b^	10.60 ± 0.49^a^
Carbohydrate (g/100 g)	5.77 ± 0.21^b^	9.36 ± 0.28^a^
Total fiber (g/100 g)	0.45 ± 0.01^b^	0.70 ± 0.03^a^
Total sugar (g/100 g)	0.89 ± 0.10^a^	0.84 ± 0.01^a^
Moisture (g/100 g)	63.68 ± 0.25^a^	60.21 ± 0.42^b^
Ash (g/100 g)	1.44 ± 0.05^a^	1.56 ± 0.02^a^
Sodium (mg/100 g)	328.08 ± 0.01^a^	324.76 ± 0.01^b^
Calcium (mg/100 g)	30.73 ± 0.65^a^	24.49 ± 0.15^b^
Cholesterol (mg/100 g)	68.42 ± 0.08^b^	89.00 ± 0.07^a^
Energy value (kcal/100 g)	180.92 ± 1.07^b^	203.55 ± 0.71^a^

Data are expressed as mean ± standard deviation (n = 3).

SP0-40 (Control): Chicken bread spread without silkworm pupae fortification using the optimum level of coconut oil obtained from experiment 2.

SP50-40: Chicken bread spread with 50% replacement of chicken by silkworm pupae using the optimum level of coconut oil obtained from experiment 2.

Different lowercase superscripts within the same row indicate significant differences (*p* ≤ 0.05).

## Conclusion

The fortification of chicken breast bread spread with SP affected the physicochemical and sensory characteristics of the final product. The increased level of SP increased the fat content and TEF (%) of SP-fortified chicken bread spread. The dark-brown color of the SP exoskeleton resulted in the SP-fortified chicken bread spread presenting a darker color compared with the control sample (without fortification). The fortification of chicken bread spread with SP at 50% (SP50) was generally accepted by the consumer. Moreover, the reduced CO level used to prepare SP-fortified chicken bread spread could reduce the TEF, whereas the denseness and resistance during spreading were increased. Reducing the CO to 40% of CO content in the control sample (SP50-40) was suitable for preparing SP-fortified chicken bread spread with satisfactory sensory properties. Based on the nutritional value, these results indicated that the SP could be used as an alternative protein and fat source for preparing bread spread, improving the added value of edible insects such as silkworm pupae.
